# Validation Evidence for the Children’s Eating Behaviour Questionnaire (CEBQ) in Brazil: A Cross-Sectional Study

**DOI:** 10.3390/nu17050851

**Published:** 2025-02-28

**Authors:** Marina Zanette Peuckert, Camila Ospina Ayala, Rita Mattiello, Thiago Wendt Viola, Marthina Streda Walker, Ana Maria Pandolfo Feoli, Caroline Abud Drumond Costa

**Affiliations:** 1 Graduate Program Pediatrics and Child Health, Pontifical Catholic University of Rio Grande do Sul (PUCRS), Ipiranga Avenue, 6681, Porto Alegre 90610-970, RS, Brazil; camila.ayala@edu.pucrs.br (C.O.A.); thiago.wendt@pucrs.br (T.W.V.); marthina.walker@acad.pucrs.br (M.S.W.); anafeoli@pucrs.br (A.M.P.F.); caroline.drumond@pucrs.br (C.A.D.C.); 2 School Medicine, Universidade Federal do Rio Grande do Sul (UFRGS), Rua Ramiro Barcelos, 2400, Porto Alegre 90010-150, RS, Brazil; rita.mattiello@ufrgs.br

**Keywords:** children’s eating behaviour questionnaire, validation study, children’s eating behavior, psychometrics, exploratory factor analysis

## Abstract

Background: Eating behavior is influenced by intrinsic and extrinsic factors from early infancy, shaping an individual’s relationship with food. Tools such as the Children’s Eating Behaviour Questionnaire (CEBQ) were designed to evaluate these patterns in children and facilitate the early identification of potential issues. Objective: The objective of this study is to validate the CEBQ for use in Brazilian children and adolescents. Methods: Parents/caregivers of students from public and private schools in southern Brazil completed the CEBQ. Anthropometric measurements of students’ weight and height were also taken. Psychometric properties were assessed by internal consistency (Cronbach’s alpha), construct validity (exploratory factor analysis), and criterion validity (correlation between CEBQ scores and participants’ body mass index-for-age categories). Results: A total of 205 participants aged 3 to 13 and their caregivers participated in this study. Exploratory factor analysis resulted in a five-factor questionnaire and a reduction of four items. All remaining items had a factor loading > 0.3. Cronbach’s alpha values were satisfactory, with values ≥ 0.7 in all factors, supporting the instrument’s internal consistency. The findings also showed significant associations between CEBQ scales and participants’ BMI for age. Conclusions: The findings of this study provide evidence that the CEBQ is a valid tool for assessing eating behavior in Brazilian children and adolescents.

## 1. Introduction

The frequent repetition of actions characterizes a habit, which extends to norms of behavior [1]. Eating behavior, however, is not limited to frequency and eating routines. It is a complex phenomenon [2], influenced by a set of internal and external factors to which individuals are exposed from early infancy, predicting how they will relate to food [1,3,4]. While eating behaviors are susceptible to changes across the lifespan, changes during adulthood are challenging. This highlights the importance of nourishing a healthy relationship with food from an early age [3].

Globally, eating disorders are a pressing public health concern, affecting approximately 23% of children and adolescents. These rates are even more alarming in Brazil, where nearly 40% of this population is affected. Such statistics highlight the urgent need for early identification and intervention to address these issues [5]. Understanding eating behavior patterns is essential for tailoring nutritional strategies to promote healthier habits and address disordered eating tendencies.

Adequate nutritional diagnosis is essential for developing strategies and approaches tailored to individual needs [6]. Within the context of research into children’s and adolescents’ eating behavior, the Children’s Eating Behaviour Questionnaire (CEBQ) was developed in London in 2001 [7]. The items used in the questionnaire were developed based on the existing literature, modified questions from existing scales, and interviews with parents and caregivers about their children’s eating behavior.

The instrument consists of 35 items to be completed by the child’s primary caregiver on a 5-point Likert scale, as follows: 1 (never), 2 (rarely), 3 (sometimes), 4 (often), and 5 (always). Eating style is assessed via eight scales: satiety responsiveness, slowness in eating, food fussiness, food responsiveness, enjoyment of food, desire to drink, emotional overeating, and emotional undereating. To assess eating behavior, it is necessary to sum up the scores of all items on the same scale and compare the results of all scales to determine which one prevails [7].

The application of the CEBQ has positively impacted the youth population by helping to address and prevent food-related complications [7]. The questionnaire is key in advancing scientific knowledge by providing relevant data contributing to multidisciplinary research on child health [7]. The CEBQ is widely used in different cultures and has been adapted and validated in several countries, including Saudi Arabia [8], Poland [9], the Netherlands [10], Ethiopia [11], and Portugal [12].

This study aimed to validate the CEBQ for use in Brazilian children and adolescents by analyzing its original eight-factor structure, reliability, and criterion validity. Such validation plays a crucial role in providing health professionals with an accurate instrument for characterizing eating patterns in this age group, allowing for better-targeted interventions and a solid foundation for future research.

## 2. Methods

### 2.1. Study Design

This cross-sectional study was conducted to validate the CEBQ and followed the recommendations of the Strengthening the Reporting of Observational Studies in Epidemiology (STROBE) statement (Supplementary File S1) [13]. Permission to use the CEBQ was obtained from the original developers.

The study was approved by the Research Ethics Committee of Pontifícia Universidade Católica do Rio Grande do Sul (approval number 5,957,048), Brazil, and followed the regulatory standards for research involving human participants (Resolution No. 466/2012 of the Brazilian National Health Council). The children and adolescents who agreed to participate in the study provided informed assent, and their parents or legal representatives provided written informed consent.

### 2.2. Participants

Participants were recruited by convenience sampling from public and private schools in southern Brazil between May and August 2023. The questionnaires were administered in person using mobile devices by approaching the children/adolescents and their parents/caregivers at the beginning and end of the school day and at weekend events. All parents/caregivers on-site were invited to participate. Pregnant adolescents were not eligible for the study.

### 2.3. Data Collected

#### 2.3.1. Children’s Eating Behavior Questionnaire (CEBQ)

The parents/caregivers of the participants included in the study were invited to complete the CEBQ, translated and adapted to Brazilian Portuguese [14].

#### 2.3.2. Sociodemographic Variables

Sociodemographic data included date of birth, sex, level of education, and self-reported skin color.

#### 2.3.3. Children’s Anthropometric Measurements

Anthropometric measurements were used as a comparison parameter for the criterion validity of the CEBQ. Body mass was measured with the participants wearing minimal clothing and no shoes on a calibrated digital scale (premium B31E-AVANUTRI). The participants’ height was measured barefoot using a portable wall-mounted stadiometer of up to 2.10 m (AVANUTRI). To obtain z-scores, the data on weight and height were transferred to the World Health Organization (WHO) Anthro 3.2.2 software for children aged 0 to 5 years and WHO AnthroPlus 1.0.4 software for children and adolescents aged 5 to 19 years [15]. Nutritional status was classified using the body mass index (BMI)-for-age and height-for-age anthropometric indicators from the WHO curves [16,17].

### 2.4. Sample Size

The sample size was determined based on the psychometric recommendation of a minimum of five participants per item (5:1 ratio), ensuring statistical reliability and robust factor analysis. The 35 items in the instrument resulted in a required sample size of 175 participants. This ratio is widely accepted in psychometric research as it balances practicality and the stability of factor solutions, provides sufficient statistical power, and minimizes variability in the results [18].

### 2.5. Psychometric Properties and Data Analysis

#### 2.5.1. Concurrent Criterion Validity

Criterion validity was established by correlating CEBQ scores and participants’ BMI-for-age scores using Spearman’s correlation due to the skewed distribution of the data.

#### 2.5.2. Construct Validity

Construct validity was determined by exploratory factor analysis (EFA). This technique aims to identify the latent factors in the instrument, more effectively representing the variables observed in the group under analysis [19]. The correlation matrix between the items was initially used to assess the feasibility of applying EFA. Bartlett’s test of sphericity was then performed, in which a significant value (<0.05) is indicative of adequacy. The Kaiser–Meyer–Olkin (KMO) index was also calculated, where values > 0.7 indicate suitability for EFA. The visual inspection of the scree plot and parallel analysis were considered for factor retention. Oblique (oblimin) rotation was used due to the internal correlation of the factor structure. The salience of item loadings on factors was determined for significant coefficients ≥ 0.3. Items with non-significant factor loadings were removed, and the analysis was re-run [19,20].

#### 2.5.3. Reliability

Reliability was assessed by internal consistency using the Cronbach’s alpha coefficient, which reflects the level of covariance between the items of a scale. Values ≥ 0.7 are considered adequate [21,22].

#### 2.5.4. Data Analysis

All analyses were performed using R Studio Statistical Software (version 4.3.2). Categorical variables are expressed as absolute and relative frequencies, while continuous variables are expressed as the mean and standard deviation (SD). For the CEBQ analysis, the mean total score of each scale and the reversed items were calculated.

## 3. Results

### 3.1. Characteristics of the Sample

A total of 205 dyads participated in the study, each consisting of a parent or caregiver and a child or adolescent. Most respondents were mothers, accounting for 77% of the sample. Most participants had a high monthly family income, above BRL 6000.00. Furthermore, almost 70% of respondents self-identified as White (Table 1).

Most children included in the study were male (52.68%), with a mean age of 7.72 (SD 2.38) years, and 71.71% were identified as White. Almost half of the children were classified as having normal weight, whereas 43.9% were classified as having excess weight (overweight, obesity, and severe obesity) (Table 2).

### 3.2. Criterion Validity

Criterion validity was determined by correlating BMI-for-age z-scores and CEBQ scores. Of the eight scales, five generated significant correlations with the z-scores (*p* < 0.05). As shown in Table 3, satiety responsiveness (rho = −0.2631, *p* = 0.0001) and slowness in eating (rho = −0.1534, *p* = 0.0279) showed a significant negative correlation with BMI-for-age scores, indicating that children with greater satiety responsiveness and slowness in eating tend to have lower BMI-for-age scores. Conversely, enjoyment of food (rho = 0.1677, *p* = 0.0162), emotional overeating (rho = 0.1619, *p* = 0.0203), and food responsiveness (rho = 0.1735, *p* = 0.0128) showed a significant positive correlation with BMI-for-age scores, suggesting that children with greater enjoyment of food, emotional overeating, and food responsiveness tend to have higher BMI-for-age scores. The food fussiness, emotional undereating, and desire to drink scales showed no significant correlation with BMI for age.

### 3.3. Exploratory Factor Analysis (EFA)

The KMO index (0.84) and the results of Bartlett’s test of sphericity (*p* < 0.01) were favorable and adequate for the acceptability of the correlation matrix between the items, supporting the continuation of EFA [23]. The parallel analysis revealed that a six-factor structure would better fit the questionnaire than the original eight-factor structure. However, the visual inspection of the scree plot showed that the retention of five factors would be more representative of the data. Therefore, EFA was continued with a five-factor solution.

**Figure 1:**
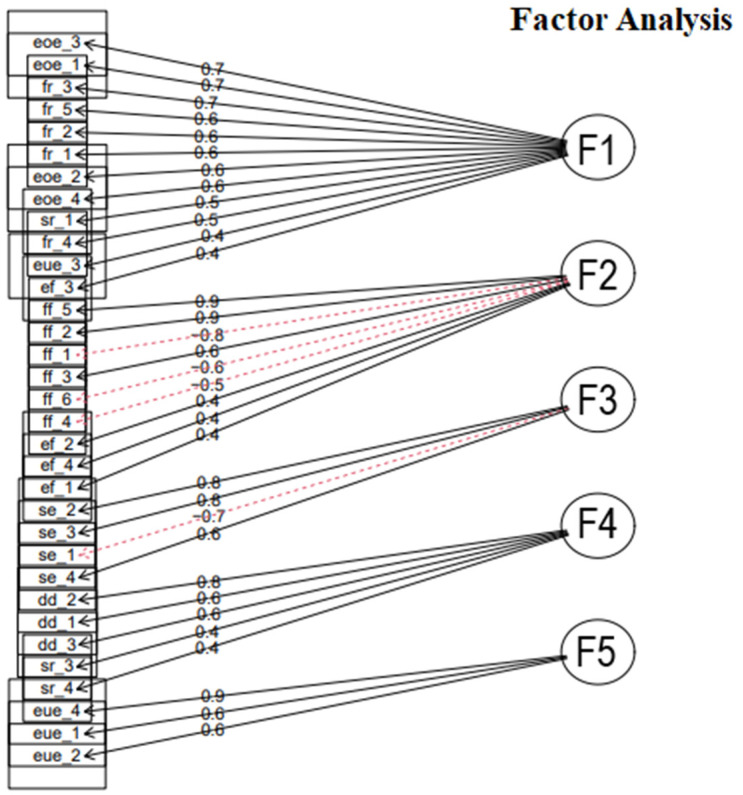
Diagram of exploratory factor analysis. Legend: F1 factor 1; F2 factor 2; F3 factor 3; F4 factor 4; F5 factor 5. The dashed line present in some questions represents the negative association, meaning that the sum of these questions should be done inversely in the total score of each scale.

Items 17 and 30 (originally from the satiety responsiveness scale) were not grouped into five scales because they had factor loadings of 0.26 and 0.21, respectively. Although a factor loading < 0.3 was a criterion for excluding items, theoretical support must also be considered. Factor 4 showed problems, since the grouped factors were the desire to drink and satiety responsiveness scales. However, to group these two scales, it would be necessary for the items in one of them to have a negative factor loading, which did not occur. Therefore, the factor loadings were re-analyzed to check if any of them were below 0.5, and the items ‘My child eats less when upset’ and ’My child gets full up easily’ achieved factor loadings of 0.38 and 0.42, respectively. Therefore, an additional EFA was conducted without these four items. The factor loadings are shown in Table 4 and Figure 1.

As the number of factors reduced with the EFA, the grouping of items changed from the original questionnaire. Factor 1 grouped items from five original scales: satiety responsiveness, enjoyment of food, emotional undereating, food responsiveness, and emotional overeating. All items corresponded to overeating, thus naming this new scale.

Factor 2 grouped items from the enjoyment of food and food fussiness scales. However, the items ’My child refuses new foods at first’, ’My child decides that s/he like a food, even without tasting it’, and ’My child is difficult to please with meals’ displayed negative factor loadings, that is, the items should be reverse scored for the total score to be interpretable because the construct associated with this factor was the enjoyment of food.

In factor 3, the grouping of items did not change from the original questionnaire. All items that form this construct belong to the slowness in eating scale. Factor 4 behaved comparably, consisting only of items from the desire to drink scale. Factor 5 included the remaining items from the emotional undereating scale. The five resulting scales were renamed to capture the latent constructs more accurately: ’overeating’, ‘enjoyment of food’, ’slowness in eating’, ’desire to drink’, and ’emotional undereating’.

### 3.4. Internal Consistency

The Cronbach’s alpha for the five factors retained in the EFA indicated an acceptable instrument. Factor 2 had the highest reliability, with a value of 0.88. Factors 1, 3, and 4 also demonstrated good levels of internal consistency, with values of 0.82, 0.78, and 0.78, respectively. This suggests quite solid reliability in the measurements of these factors. However, factor 5 demonstrated acceptable consistency, with a value of 0.70, indicating greater variability in the responses to the items associated with this factor (Table 5).

## 4. Discussion

The present study aimed to validate the five-factor structure CEBQ for use in Brazilian children and adolescents, demonstrating adequate psychometric properties when examining the resulting five-factor structure. These findings support the instrument’s reliability and validity for assessing eating behavior traits within this population, contributing to its potential use in both clinical and research settings to better understand and address eating behaviors.

To date, this has been the only study to demonstrate the better performance of the instrument with five factors. The literature shows that the questionnaire performs differently in countries with eight-, seven-, and six-factor structures [8,24,25]. Cultural differences between countries may generate these differences in the reproduction of CEBQ results since people’s place of residence has a major influence on their eating behavior [4]. Other variables can also explain the variation in the number of factors. The study by Manzano et al. (2021) [26]. resulted in a three-factor questionnaire, but their sample consisted of school-age children who were overweight and obese. This outcome is possible because nutritional status also correlates with eating behavior [8,27].

The correlations between CEBQ scores and participants’ BMI-for-age scores were significant on some scales, which is consistent with previous studies that used the same analysis model. The enjoyment of food and overeating scales, linked to interest in food, were positively associated with higher levels of BMI-for-age scores. Different studies have found similar food scale enjoyment results in countries such as Saudi Arabia, Chile, Portugal, and the Netherlands [8,10,27,28]. The satiety responsiveness scale was inversely associated with BMI-for-age scores, supporting the view that regulatory capacity may be impaired in people with higher weight [29].

In validation studies of the CEBQ in other countries [9,11], the Cronbach’s alpha for the food responsiveness and slowness in eating scales was considered poor to questionable. The five-factor structure of the CEBQ for the Brazilian population yielded more favorable results in all factors, indicating that the reliability of an instrument is not a constant feature, being somewhat variable in different environments and situations [22].

Data collection revealed disparities in the family income and education level of most parents/caregivers who participated in the study compared to the real Brazilian scenario. According to the 2020 Brazilian National Education Plan, the average education level of Brazilians is 10.2 years, which is nested within the category of incomplete high school [30]. The average monthly family income in Brazil from the third quarter of 2022 to the first and second quarters of 2023, when the data were collected, did not exceed a 1.5 minimum monthly salary, according to the Brazilian Institute of Geography and Statistics [31]. Of note, the Brazilian minimum monthly salary was BRL 1320.00 in 2023 and denotes government regulation for a minimum monthly rate paid for a worker who works, on average, 44 h a week for 4 weeks a month. Although data collection aimed to ensure that the study would replicate the Brazilian context as much as possible, the rate of participants from private schools was higher.

The present study has limitations that need to be addressed. The sample size did not allow confirmatory factor analysis to determine possible adjustments required for this version. The generalizability of the findings from this study is a potential limitation that must be acknowledged. While all participants were recruited in Rio Grande do Sul, the authors made efforts to mitigate this limitation by expanding the research to include a diverse sample of children and adolescents from various socioeconomic and ethnic groups, aiming to better represent the Brazilian population. These efforts strengthen the study’s applicability, but regional differences within Brazil may still influence the findings. Future research should include samples from other regions of the country to further enhance generalizability and provide a more comprehensive understanding of the studied phenomenon. Another study limitation is that criterion validity was not assessed using a gold-standard tool. However, this is the main reason we are validating the CEBQ, as no instruments are available to validate this latent trait in our population. In addition, BMI is a commonly used alternative for correlations in this type of assessment. The satisfactory performance of the psychometric properties evaluated in the overall results is a strong indicator of validity. However, further assessments of the items, such as the application of Item Response Theory, could complement and enhance this evaluation.

The data collected from Brazilian children failed to replicate the original eight-factor structure of the CEBQ. However, the resulting five-factor structure with 31 items fit the data well. Previous validation studies of the CEBQ in distinct cultures also needed to remove items due to low factor loadings [8,32,33].

## 5. Conclusions

The findings of this study demonstrate the validity and reliability of the CEBQ to assess the eating behavior of children and adolescents in Brazil. The analyses showed that a five-factor structure can explain the total variance of the instrument. Furthermore, our findings showed a significant association between two scales linked to interest in food and children having higher BMIs for age. Consistent with these results, three scales related to food refusal had a negative association with BMI-for-age scores. Incorporating this tool into the nutritional assessment of children and adolescents will be of great importance in better investigating eating behavior in this age group.

## Figures and Tables

**Table 1:** Distribution of parent/caregiver characteristics (N = 205).

Variable	n (%)
***Level of education***	
No schooling completed	2 (0.98)
Incomplete elementary school	25 (12.20)
Complete elementary school	8 (3.90)
Incomplete high school	21 (10.25)
Complete high school	12 (5.85)
Incomplete technical degree	1 (0.50)
Complete technical degree	6 (2.92)
Incomplete college degree	7 (3.41)
Complete college degree	44 (21.46)
Graduate degree (beyond bachelor’s degree)	79 (38.53)
***Monthly family income (R$)***	
Up to 300.00	7 (3.41)
301.00–500.00	6 (2.93)
501.00–800.00	17 (8.29)
801.00–1212.00	24 (11.70)
1213.00–2424.00	16 (7.80)
2425.00–3636.00	8 (3.90)
3637.00–4848.00	2 (0.99)
4849.00–6060.00	17 (8.30)
>6061.00	108 (52.68)
***Kinship***	
Mother	159 (77.56)
Father	33 (16.09)
Grandmother	6 (2.92)
Grandfather	3 (1.47)
Uncle/aunt	4 (1.96)
***Race/skin color***	
White	138 (67.31)
Black	43 (20.97)
Yellow (Asian descent)	2 (0.98)
Indigenous	1 (0.49)
Brown (mixed race)	21 (10.25)

n, number, %, percentage, (R$), real.

**Table 2:** Characteristics of children and adolescents (N = 205).

Variable	Result
Age (years), mean (SD)	7.72 (2.38)
***Sex, n (%)***	
Female	97 (47.32)
Male	108 (52.68)
***Skin color, n (%)***	
White	147 (71.71)
Black	33 (16.10)
Yellow (Asian descent)	2 (0.97)
Indigenous	0 (0.0)
Brown (mixed race)	23 (11.22)
***Nutritional status (z-score), n (%)***	
Severe thinness	1 (0.49)
Thinness	2 (0.98)
Normal weight	96 (46.83)
Risk of overweight	16 (7.80)
Overweight	54 (26.34)
Obesity	24 (11.71)
Severe obesity	12 (5.85)
Weight (kg), mean (SD)	30.12 (10.93)
Height (cm), mean (SD)	126.03 (17.61)
BMI-for-age, mean (SD)	1.02 (1.35)
Height-for-age, mean (SD)	0.21 (1.11)

BMI, body mass index, SD, standard deviation, n, number, %, percentage, kg, kilogram, cm, centimeter.

**Table 3:** Correlation between CEBQ and BMI-for-age scores.

Scale	rho	*p*-Value
Satiety responsiveness	−0.2631	0.0001 *
Slowness in eating	−0.1534	0.0279 *
Enjoyment of food	0.1677	0.0162 *
Food fussiness	−0.0339	0.6289
Emotional undereating	−0.0774	0.2695
Desire to drink	0.0160	0.8189
Emotional overeating	0.1619	0.0203 *
Food responsiveness	0.1735	0.0128 *

CEBQ, Children’s Eating Behaviour Questionnaire; BMI, body mass index; rho, Spearman’s correlation coefficient. * *p*-value significant at < 0.05.

**Table 4:** Factor loadings of items from the third exploratory factor analysis.

Item	Factor 1	Factor 2	Factor 3	Factor 4	Factor 5
My child eats more when anxious.	0.75				
My child eats more when worried.	0.67				
Given the choice, my child would eat most of the time.	0.67				
If given the chance, my child would always have food in his/her mouth.	0.64				
If allowed to, my child would eat too much.	0.63				
My child is always asking for food.	0.59				
My child eats more when annoyed.	0.59				
My child eats more when s/he has nothing else to do.	0.57				
My child has a big appetite.	0.51				
Even if my child is full up, she/he finds room to eat his/her favorite food.	0.48				
My child eats more when s/he is happy.	0.43				
My child looks forward to mealtimes.	0.41				
My child is interested in tasting food she/he hasn’t tasted before.		0.88			
My child enjoys tasting new foods.		0.87			
My child refuses new foods at first.		−0.75			
My child enjoys a wide variety of foods.		0.65			
My child decides that s/he doesn’t like a food, even without tasting it.		−0.61			
My child is difficult to please with meals.		−0.53			
My child is interested in food.		0.43			
My child enjoys eating.		0.42			
My child loves food.		0.37			
My child eats slowly.			0.77		
My child takes more than 30 min to finish a meal.			0.75		
My child finishes his/her meal quickly.			0.71		
My child eats more and more slowly during a meal.			0.57		
If given the chance, my child would drink continuously throughout the day.				0.78	
My child is always asking for a drink.				0.64	
If given the chance, my child would always be having a drink.				0.56	
My child eats less when upset.					0.86
My child eats less when angry.					0.58
My child eats less when she/he is tired.					0.55

**Table 5:** Internal consistency analysis after exploratory factor analysis.

Factor	Cronbach’s Alpha
Overeating	0.82
Enjoyment of food	0.88
Slowness in eating	0.78
Desire to drink	0.78
Emotional undereating	0.70
Total	0.79

## Data Availability

The data presented in this study are available on request from the corresponding author due to the privacy of the database.

## Supplementary Materials

The following supporting information can be downloaded at: https://www.mdpi.com/article/10.3390/nu17050851/s1, File S1: Check-list STROBE.
